# Satellite gravity measurement monitoring terrestrial water storage change and drought in the continental United States

**DOI:** 10.1038/srep19909

**Published:** 2016-01-27

**Authors:** Hang Yi, Lianxing Wen

**Affiliations:** 1 Laboratory of Seismology and Physics of Earth’s Interior; School of Earth and Space Sciences, University of Science and Technology of China, Hefei, Anhui, 230026, P. R. China; 2 Department of Geosciences, State University of New York at Stony Brook, Stony Brook, NY 11794, USA

## Abstract

We use satellite gravity measurements in the Gravity Recovery and Climate Experiment (GRACE) to estimate terrestrial water storage (TWS) change in the continental United States (US) from 2003 to 2012, and establish a GRACE-based Hydrological Drought Index (GHDI) for drought monitoring. GRACE-inferred TWS exhibits opposite patterns between north and south of the continental US from 2003 to 2012, with the equivalent water thickness increasing from −4.0 to 9.4 cm in the north and decreasing from 4.1 to −6.7 cm in the south. The equivalent water thickness also decreases by −5.1 cm in the middle south in 2006. GHDI is established to represent the extent of GRACE-inferred TWS anomaly departing from its historical average and is calibrated to resemble traditional Palmer Hydrological Drought Index (PHDI) in the continental US. GHDI exhibits good correlations with PHDI in the continental US, indicating its feasibility for drought monitoring. Since GHDI is GRACE-based and has minimal dependence of hydrological parameters on the ground, it can be extended for global drought monitoring, particularly useful for the countries that lack sufficient hydrological monitoring infrastructures on the ground.

The Gravity Recovery and Climate Experiment (GRACE) twin satellites were launched in March 2002 and measure the Earth’s gravity fields every month at the global scale^1^,^2^,^3^,^4^. The monthly measurements have provided new understanding of mass redistributions within the Earth’s system, including terrestrial water storage (TWS) change^1^,^2^,^3^,^4^,^5^,^6^,^7^,^8^,^9^,^10^,^11^,^12^,^13^,^14^,^15^, mass changes associated with polar ice sheet melting^16^,^17^,^18^,^19^,^20^,^21^,^22^,^23^,^24^ and mountain glacial change^25^,^26^,^27^, surface movement associated with the glacial isostatic adjustment (GIA)^28^,^29^, non-steric sea level and ocean mass changes^30^,^31^,^32^,^33^,^34^, mass/density changes related to the co- and post-seismic processes of earthquake^35^,^36^,^37^,^38^,^39^, and mass change in the Earth’s core^40^.

Another potential application of GRACE data is drought monitoring in the continental regions^41^,^42^,^43^,^44^,^45^. Drought is a continuous period when a region receives a deficiency in its water supply, and can cause significant damage and harm to the local ecosystem and agriculture. At present, Palmer Hydrological Drought Index (PHDI) is the most prominent index of hydrological drought used in the United States (US)^46^, which is first introduced by Palmer to assess long-term moisture supply in a region^47^. However, PHDI does not take into account of man-made changes such as increased irrigation, new reservoirs, and added industrial water. It mainly reflects the moisture anomaly in the shallow surface. Besides, computation of PHDI is complex and requires inputs of several hydrological variables, which restricts PHDI to be a global drought index. Since GRACE provides observation on a global scale and GRACE-inferred TWS includes soil moisture and groundwater, it may be desirable for global drought monitoring if a similar GRACE-based hydrological drought index can be established with minimal dependence on ground hydrological data.

In this study, we use GRACE observations to infer TWS change in the continental US in the period from 2003 to 2012, and discuss the comparison between GRACE-inferred TWS and ground observations of soil moisture and groundwater at 12 locations where ground hydrological data are available. We then present a GRACE-based Hydrological Drought Index (GHDI) and its comparison with traditional PHDI in the continental US.

## Results and Discussion

### GRACE-inferred TWS change

**Figure 1:**
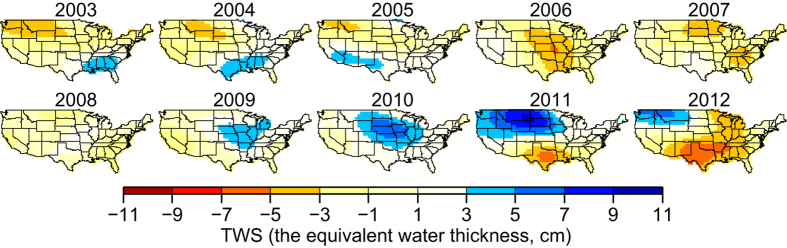
Yearly Relative GRACE-inferred TWS. Yearly averages of GRACE-inferred TWS in the continental US, in reference to their long-term mean from 2003 to 2012. The maps were created using the Generic Mapping Tools software package 4.5.12.

We use GRACE data to infer yearly relative TWS averages in the continental US in reference to their long-term mean in the period from 2003 to 2012 (Fig. 1, see Methods and Supplementary Note 1 for details). The yearly relative GRACE-inferred TWS reveals nearly opposite patterns between the north and south continental US in the period from 2003 to 2012, with a general trend of increase in the north and decrease in the south. In the north, the estimated equivalent water thickness increases from −4.0 cm in 2003 to 9.4 cm in 2011 and to 5.5 cm in 2012. In the south, the equivalent water thickness decreases from 4.1 cm in 2003 to −6.7 cm in 2012 (note that 2011 Texas drought^12^ is a drought event during the decreasing pattern in the south). Besides, there is a significant decrease of TWS in the middle south in 2006, with the maximal value of equivalent water thickness reaching −5.1 cm.

**Figure 2:**
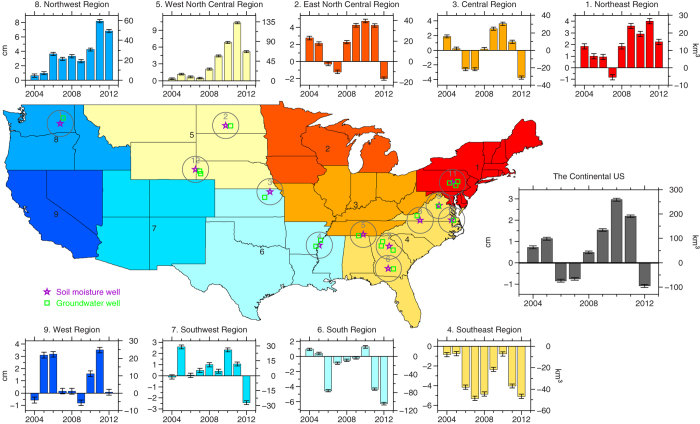
GRACE-inferred TWS change. GRACE-inferred TWS changes (in reference to 2003) with their associated uncertainties (estimated based on the root-mean-square (RMS) variations, see Supplementary Note 1 for details) in 9 hydrological divisions of the continental US and in entire region of the continental US in the period from 2004 to 2012. TWS changes are presented in both equivalent water thickness (cm, on the left side of vertical axis) and volume (km^3^, on the right side of vertical axis). Region division follows Karl and Koss^59^ and is color-coded the same way in both the panels of TWS change and the map. The gray locations labeled by the numbers are 12 locations where comparison between relative GRACE-inferred TWS and ground hydrological measurements are presented in Supplementary Fig. S1. The map was created using the Generic Mapping Tools software package 4.5.12.

We also present GRACE-inferred TWS changes in reference to 2003 and their associated uncertainties (see Supplementary Note 1 for details) in 9 hydrological divisions of the continental US and in the entire continental US, in the period from 2004 to 2012 (Fig. 2). TWS changes exhibit consistent increase in the study period with maximum of 59 km^3^ (equivalent water thickness of 8.2 cm) in northwest region in 2011 and of 133 km^3^ (equivalent water thickness of 10.3 cm) in west north central region also in 2011. On the other hand, TWS change in southeast region shows consistent decrease with two maxima in 2007 (−49 km^3^, or equivalent water thickness of −5.3 cm) and 2012 (−47 km^3^, or equivalent water thickness of −5.1 cm) respectively. Besides, TWS changes exhibit increase in northeast, east north central, west and southwest regions, in most years of the study period, with exception of a few years (northeast region in 2007, east north central region in 2006, 2007 and 2012, west region in 2004 and 2009, and southwest region in 2004 and 2012), and decrease in south region in most years of the study period, with exception of 2004, 2005 and 2010, while it fluctuates between −30 to 31 km^3^ (equivalent water thickness from −3.6 to 3.7 cm) in central region. Overall, TWS change exhibits increase in the continental US in most years of the study period with the maximum increase of 259 km^3^ (equivalent water thickness of 3.0 cm) in 2010, with exception of decrease in 2006, 2007 and 2012.

We compare relative GRACE-inferred TWS (in reference to their long-term mean from 2003 to 2012) to ground measurements at 12 different locations in the continental US where ground hydrological data are available (gray locations labeled by the numbers in Fig. 2) in the period from 2003 to 2012 (see Supplementary Fig. S1, Supplementary Note 2 and Supplementary Discussion for details). GRACE-inferred TWS exhibits good correlation (correlation coefficients greater than 0.6, Supplementary Table S1) and similar trend and annual cycle comparing to the inverted ground-based TWS, which provides further validation of GRACE-inferred TWS. Comparisons with ground data also suggest that the relative contributions of soil moisture and groundwater to TWS change vary from region to region across the continental US. Soil moisture plays the dominant role in TWS change at some locations of northwest and central regions of the continental US (gray locations 1 and 3 in Fig. 2); groundwater greatly contributes to TWS change at some locations of north central and east regions of the continental US (gray locations 2, 7, 8 and 11 in Fig. 2); and both soil moisture and groundwater contribute to TWS change at other locations (Supplementary Table S1). These relatively different contributions to TWS change between groundwater and soil moisture in different regions are likely related to hydrological conditions, especially the type of soil deposit, in the region. For example, regions that have thick soil deposits would have high water-retaining capacity in the soils and relatively stable groundwater table. As result, the change of soil moisture storage constitutes most of the TWSA in those regions. On the other hand, in the regions that have thin soil deposits, water-retaining capacity in the soils is low and groundwater table is less stable. As result, the fluctuation of groundwater table would contribute a large portion of the TWSA in those regions.

### GRACE-based Hydrological Drought Index (GHDI)

We establish a hydrological drought index based on GRACE observations, named GRACE-based Hydrological Drought Index (we term it GHDI). GHDI is defined in a simpler form, but maintains similar concepts in PHDI’s original definition (see Supplementary Note 3 for details). The definition follows these two principles: 1) GHDI should resemble PHDI used in the traditional drought monitoring and 2) GHDI should rely on GRACE observations with minimal dependence on hydrological parameters of the region. To fulfill that goal, an empirical relationship between GHDI values and GRACE observations is needed. We calibrate the empirical relationship using GRACE observations and PHDI values in the continental US, as the region has a well-established PHDI monitoring history and extensive record of soil moisture.

We define GHDI as an indicator of the extent of GRACE-inferred TWS anomaly in a region departing from its historical average, i.e.,


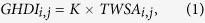


where *i* is for year, *j* for month, *K* the converting factor which varies from region to region, and *TWS*_*i*, *j*_ GRACE-inferred TWS anomaly for the *j* th month of year *i*, i.e.,


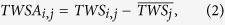


where *TWS*_*i*, *j*_ is GRACE-inferred TWS for the *j*th month of year *i*, and 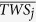 the long-term mean (from 2003 to 2012) of GRACE-inferred TWS of the same month (the *j*th month of a year). We define *K* as:


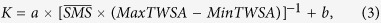


where 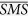 is the long-term mean of soil moisture storage (SMS) of the region (from 2003 to 2012, see Supplementary Note 4 for details), *MaxTWSA*-*MinTWSA* represents the magnitude of historical variation of *TWSA* of the region (from 2003 to 2012), and *a*, *b* are proportional constants. The rational of this definition is such that GHDI represents the percentage of soil moisture anomaly in a particular region (i.e., inversely proportional to the mean of soil moisture of the region), with its scale normalized by the range of historical *TWSA* of the region.

**Figure 3:**
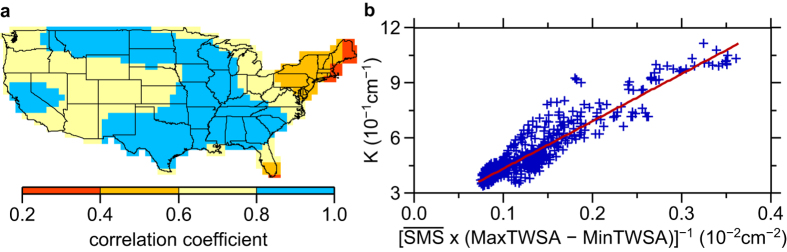
Establishing GHDI. (**a**) Correlation coefficients between the time series of GRACE-inferred TWSA and PHDI values in the continental US. Light blue region is where GRACE-inferred TWSA and PHDI values are used to infer the empirical relationship between *K* and 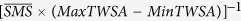 in Fig. 3b. (**b**) The inferred *K* values vs. values of 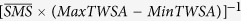 (blue crosses) in the light blue region in Fig. 3a, along with their best fitting linear curve (red line, eq. (6)). The map was created using the Generic Mapping Tools software package 4.5.12.

We attempt to make GHDI to be as close to PHDI as possible, and adopt an empirical approach to determine the values of *a* and *b*, by searching for the best-fitting parameters of *a* and *b* so that inferred GHDI values would be most close to current PHDI values adopted in the continental US. In another word, we calibrate these proportional constants using GRACE observations and PHDI values in the continental US. In practice, we calibrate GHDI using PHDI values in the regions where PHDI values exhibit good correlation with GRACE-inferred TWS anomaly (light blue regions in Fig. 3a). We choose those regions based on the assumption that soil moisture in the regions contributes most of TWSA and PHDI captures best the terrestrial water storage change there. PHDI values in other regions are used to check the consistency and difference between the two hydrological indexes. The detailed procedure of calibration is presented in Methods Establishing GHDI.

**Figure 4:**
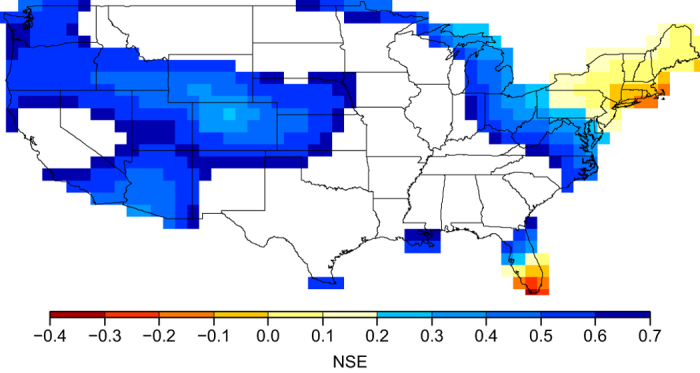
NSE values between PHDI and GHDI. Values of NSE are calculated based on Supplementary equation (S11) in the time period from 2003 to 2012 (see Supplementary Note 5 for details) in the continental US where PHDI values are not used for the calibration (excluding light blue region in Fig. 3a), with the reported monthly PHDI values considered as the observation and the calculated monthly GHDI values the prediction. Long-term means are removed in both GHDI and PHDI. The map was created using the Generic Mapping Tools software package 4.5.12.

Although GHDI is calibrated using PHDI in only some regions of the continental US (see Methods, Establishing GHDI for details), it exhibits strong similarities with PHDI in other regions of the continental US where PHDI values are not used for the calibration. Note that, values of coefficient of efficiency defined by Nash and Sutcliffe (Nash-Sutcliffe efficiency, NSE)^48^,^49^,^50^,^51^, which is a measure of hydrological model’s performance (see Supplementary Note 5 for details), are positive between monthly PHDI and GHDI values in most regions of the continental US where PHDI values are not used for the calibration, with negative values only in few regions (less than 4.0%, with the minimum value of −0.34) (Fig. 4). The locations where NSE values are below 0.2 are in the northeast region of the US and south Florida. These low values may be due to several reasons: 1) GIA correction in GRACE observations may have large uncertainties in the northeast region of the continental US and 2) quantification of groundwater contribution in the PHDI estimate of these two regions may have large uncertainties (e.g., gray location 11 in Fig. 2, see Supplementary Discussion for details). On the other hand, both of the two regions exhibit small yearly TWS changes (Fig. 1). These two factors make the quantification challenging for both indexes. The NSE values in other regions (where PHDI values are not used for calibration) outside the northeast region of the continental US and south Florida are between 0.2 and 0.65, with the average value of 0.53. We suggest that the above comparisons indicate the validity of GHDI for drought monitoring, but further improved quantification of GRACE signals from other geophysical processes, such as GIA, would be desirable.

**Figure 5:**
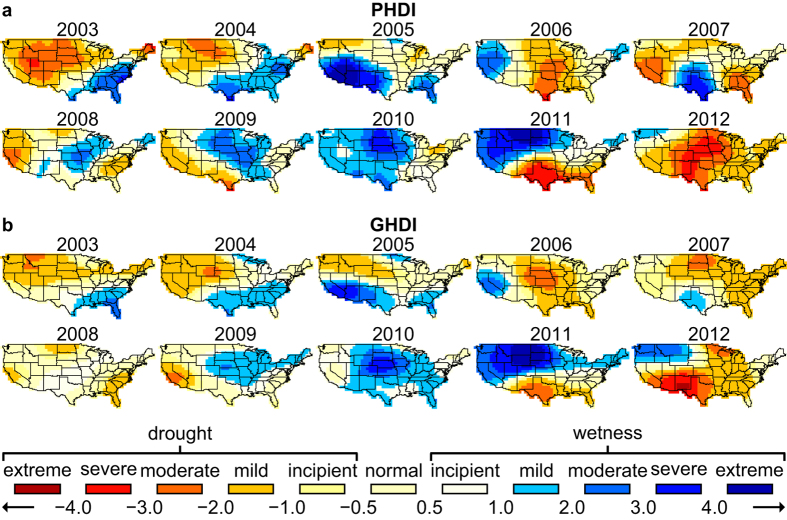
Yearly averaged PHDI and GHDI values. Yearly averages of (**a**) PHDI and (**b**) GHDI values in the continental US in the period from 2003 to 2012, with their long-term means in the study period removed respectively. The maps were created using the Generic Mapping Tools software package 4.5.12.

Strong consistency is also observed between the yearly averages of PHDI and GHDI values in the continental US from 2003 to 2012 (Fig. 5a,b). Most dry/wet index levels (classification in Table 1 47 and Supplementary Note 3) are similar between the two indexes in most regions and in most time periods. Both indexes exhibit similar geographical patterns: a dry west region and a wet east region in 2003, 2004 and 2009, a dry northwest region and a wet south region in 2005, a dry south central region and a wet west region in 2006, a dry west and southeast region and a wet south central region in 2007 and 2008, a dominantly wet continental US in the period of 2010, a dry south region and a wet north region in 2011, and a dominantly dry continental US except a dry northwest region in 2012. Exceptions lie in south central region in 2007, where PHDI records maximum to severe wet while GHDI just indicates mild wet in a smaller region, in central region in 2008, where PHDI records maximum to moderate wet while GHDI just indicates incipient wet, and in south region in 2011, where PHDI records maximum to extreme dry while GHDI just indicates moderate dry. Another noticeable difference between the two indexes lies in northeast in 2006, when GHDI and PHDI present opposite wetness/drought. The comparison between GHDI and PHDI reveals consistent yearly trend and similar amplitude. Overall, the continental US shows a wetter north region and a drier south region.

GHDI, just as GRACE observations, reflects the effects of water storage anomaly in both soil moisture and groundwater, while PHDI was derived based on hydrological balance of various hydrological variables and may not account well for all the contributions to the anomaly (see Supplementary Note 3 for details). In this regard, GHDI would reflect more accurate representation of drought condition in a region, as measurements of some hydrological variables may contain large errors in PHDI calculation.

Other part of differences between the two indexes could be attributed to different definitions of the two indexes and regional dependence of hydrological history. But overall, GHDI captures long-term drought condition and variation trend well as PHDI, and their differences are minor. GHDI computation is simple and straightforward, reflects both surface water and groundwater anomaly, and can be extended for drought monitoring on a global scale. The perspective of this possible extension would be particularly attractive, as not many countries possess the extensive hydrological monitoring system and detailed hydrological history as the US does.

## Conclusions

We estimate terrestrial water storage (TWS) change in the continental US from 2003 to 2012, based on GRACE observations. Relative GRACE-inferred TWS exhibits an increase pattern in the north of the continental US with equivalent water thickness changing from −4.0 to 9.4 cm, and a decrease pattern in the south with equivalent water thickness changing from 4.1 to −6.7 cm. A significant TWS decrease is also observed in the middle south in 2006 with the maximal value of equivalent water thickness reaching −5.1 cm. With exceptions in 2006, 2007 and 2012, the total TWS in the continental US increases in most years from 2004 to 2012 (in reference to 2003), with the maximum value of 259 km^3^ (equivalent water thickness of 3.0 cm) reaching in 2010. GRACE-inferred TWS exhibits strong similarities with the inverted ground-based TWS at 12 locations in the continental US that have ground measurements in the study period, providing further validation of GRACE-based inferences. Comparison between GRACE-inferred TWS and ground measurements also shows that soil moisture and groundwater contribute differently to TWS change in different regions.

We establish a GRACE-based Hydrological Index (GHDI) for drought monitoring. GHDI is defined as an indicator of the extent of GRACE-inferred TWS anomaly in a region departing from its historical mean and is calibrated by traditional PHDI values in the continental US from 2003 to 2012. Although GHDI is calibrated by PHDI in only some regions of the continental US, it also exhibits strong similarities with PHDI in other regions of the continental US and captures main variation trend in all of the continental US, indicating the feasibility of using GHDI to monitor drought condition and long-term variation trend in the continental areas. GHDI uses TWS anomaly contributed by both soil moisture and groundwater as the indicator and GHDI would be a better indicator of drought condition than PHDI in the regions where significant errors of hydrological variables exist. In addition, GHDI has a simpler form of calculation with minimal dependence of hydrological parameters on the ground, and can be extended for drought monitoring on a global scale, especially in the countries where the hydrological monitoring infrastructure is lacking.

## Methods

### Estimating TWS based on GRACE data

A two-step data processing is employed to suppress the noise in high degree and order spherical harmonics of the GRACE gravity solutions, following the approaches of Swenson and Wahr^52^ and Chen *et al.* 36. The monthly spherical harmonics are processed using a decorrelation filter and Gaussian smoothing. The decorrelation filter is performed such that, for a given spherical harmonics order (6 and above), a third-order polynomial function is derived by a least-squares fit to the even or odd coefficient pairs of the GRACE solutions and is removed from the monthly solutions^36^. The Gaussian smoothing^1^,^53^ is applied using a radius of 500 km. We further remove the gravity anomalies associated with the GIA from the GRACE solutions based on a geodynamical model by Paulson *et al.*^54^.

We use GRACE solutions to infer water mass in terms of equivalent water thickness^1^ and attribute them to GRACE-inferred TWS. Equivalent water thickness Δ*h*(*θ*,*φ*) is calculated as follows:


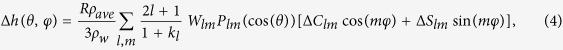


where *l* and *m* are spherical harmonics degree and order, *R* the radius of the Earth, *ρ*_*ave*_ and *ρ*_*w*_ the average density of the Earth (5517 kg/m^3^) and density of water (1000 kg/m^3^), *θ* and *φ* the colatitude and longitude, *k*_*l*_ the load Love number^1^, *W*_*lm*_ an expression in spherical harmonics for a Gaussian smoothing filter^1^, *P*_*lm*_ the normalized associated Legendre function, and Δ*C*_*lm*_ and Δ*S*_*lm*_ the normalized spherical harmonics coefficients after processed by the decorrelation filter. Degree *l* = 1 coefficients and Δ*C*_20_ are not used in the calculations, and a maximum spherical harmonics degree of 60 is used (see Supplementary Note 1 for details)^55^. The amplitude of Δ*h* is further adjusted so that the total amplitude of Δ*h* after decorrelation and smoothing filter is the same with that of initial inputs. GRACE-inferred TWS has a spatial resolution of 1° × 1° and a temporal resolution of one month.

### Establishing GHDI

We derive an empirical relationship based on GRACE-inferred *TWSA* (TWS anomaly) and *PHDI* values in the regions of the continental US where the time series of these two variables have high correlations (light blue regions in Fig. 3a, see Supplementary Note 6 and Supplementary Fig S2 for more discussions). For each climate division in the correlated region, we compute 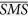 from Mosaic LSM^56^,^57^,^58^, infer *TWSA* from GRACE data, and calculate *MaxTWSA*-*MinTWSA* based on *TWSA* values, from 2003 to 2012. We estimate the best fitting *K* value in each climate region through linear-square fitting the time series of *TWSA* and *PHDI* of the region through this relationship:


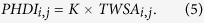


The inferred *K* values exhibit an approximately linear relationship with 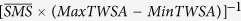 by a linearity of 0.92 and root mean square error (RMSE) of 0.065 cm^−1^ (Fig. 3b, see discussions on alternative ways of defining GHDI in Supplementary Note 7, Supplementary Fig S3a,b and Supplementary Fig. S4a,b). Such linearity indicates that GRACE observations can be used to define a hydrological index that would closely approximate PHDI. The best fitting relationship in Fig. 3b is found to be as follows (unit: 10^−1^ cm^−1^):


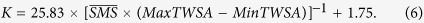


Equation (1) and (6) are the basis for calculating GHDI. We use GRACE observations, 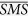 and equations (1) and (6) to calculate GHDI.

Note that, PHDI values are used to calibrate coefficients *a* and *b*. However, once these coefficients are calibrated, PHDI values are no longer needed for GHDI calculations in the future or in other regions. The only hydrological parameter needed for GHDI calculations is the mean of soil moisture storage 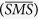 in the region.

In equation (6), *MaxTWSA*-*MinTWSA* is calculated during the period of the current time scale of GRACE mission (10 years), shorter than that of hydrological variables used in defining PHDI (27 to 71 years)^47^. In effect, we have assumed that 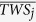 averaged over the time scale of current GRACE mission is a good representation of the long-term mean and that *MaxTWSA*-*MinTWSA* reflects the range of historic *TWSA* well. The good correlations between two indexes in the continental US suggest such assumption may be valid (see Supplementary Note 7 for more discussions). However, we suggest that such assumption should be revisited in the future as GRACE mission proceeds.

## Additional Information

**How to cite this article**: Yi, H. and Wen, L. Satellite gravity measurement monitoring terrestrial water storage change and drought in the continental United States. *Sci. Rep.* **6**, 19909; doi: 10.1038/srep19909 (2016).

## Supplementary Material

Supplementary Information

## Figures and Tables

**Table 1:** Classification of PHDI values.

PHDI value	PHDI category
4.00 and above	Extreme wetness
3.00 to 3.99	Severe wetness
2.00 to 2.99	Moderate wetness
1.00 to 1.99	Mild wetness
0.50 to 0.99	Incipient wetness
0.49 to −0.49	Normal
−0.50 to −0.99	Incipient drought
−1.00 to −1.99	Mild drought
−2.00 to −2.99	Moderate drought
−3.00 to −3.99	Severe drought
−4.00 and below	Extreme drought
