# Supplementary material for: Satellite gravity measurement monitoring terrestrial water storage change and drought in the continental United States
Source: Sci Rep. 2016 Jan 27;6:19909. doi: 10.1038/srep19909 (PMC4728606; doi:10.1038/srep19909)
Supplement: Supplementary Information [file srep19909-s1.pdf]

Supplementary Information

Satellite gravity measurement monitoring terrestrial water storage change and drought in the continental United States

Hang Yi[1,*] and Lianxing Wen[2,1]

[1]Laboratory of Seismology and Physics of Earth's Interior; School of Earth and Space Sciences, University of Science and Technology of China, Hefei, Anhui, 230026, P. R. China; and [2]Department of Geosciences, State University of New York at Stony Brook, Stony Brook, NY 11794, USA

[*]Corresponding Author

Telephone: +(86)15856943569

Email: yihang@mail.ustc.edu.cn

Supplementary Figures

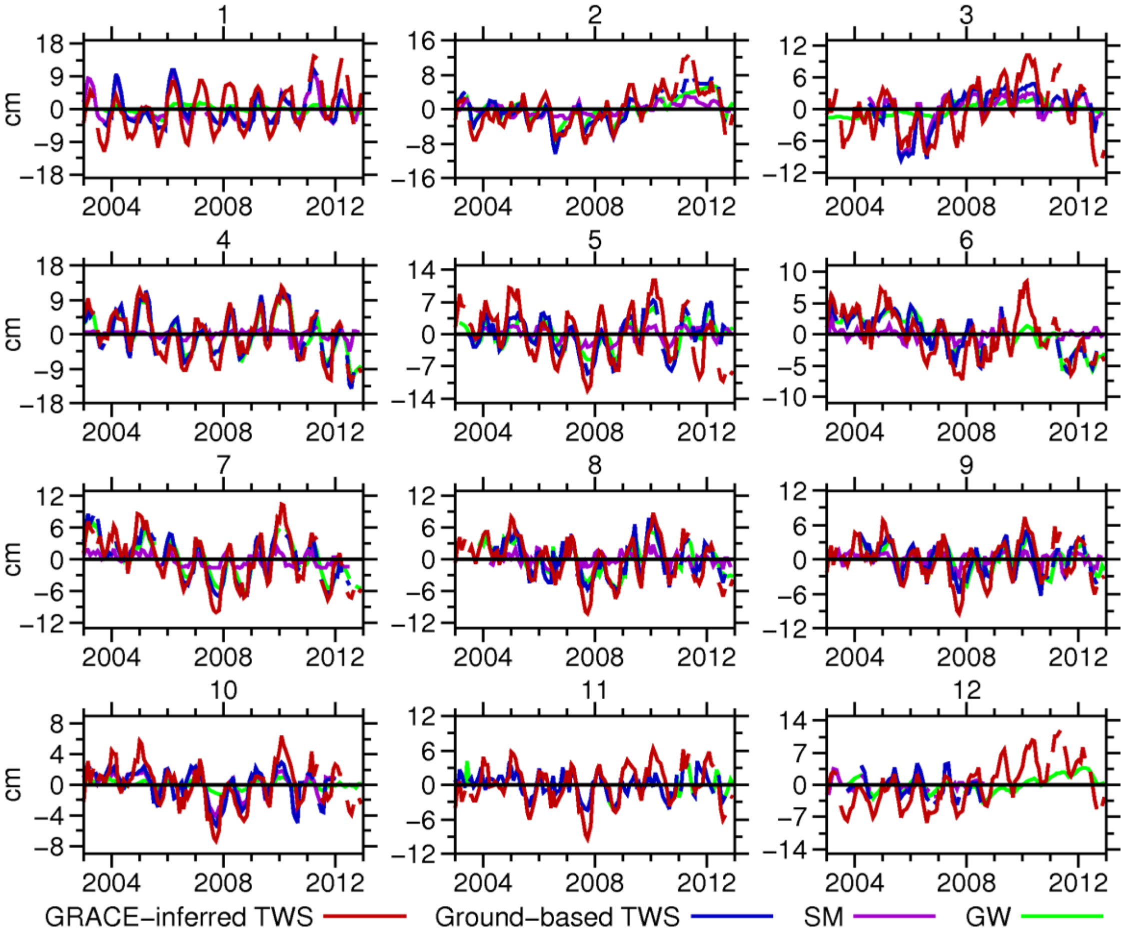

Supplementary Figure S1. Time series of relative GRACE-inferred TWS and ground observations. Time series of relative GRACE-inferred TWS (red, in reference to their long-term mean from 2003 to 2012), the inverted ground-based TWS (blue), soil moisture (purple) and groundwater (green), for 12 locations in the continental US, with panel numbers corresponding to the gray locations labeled in Fig. 2.

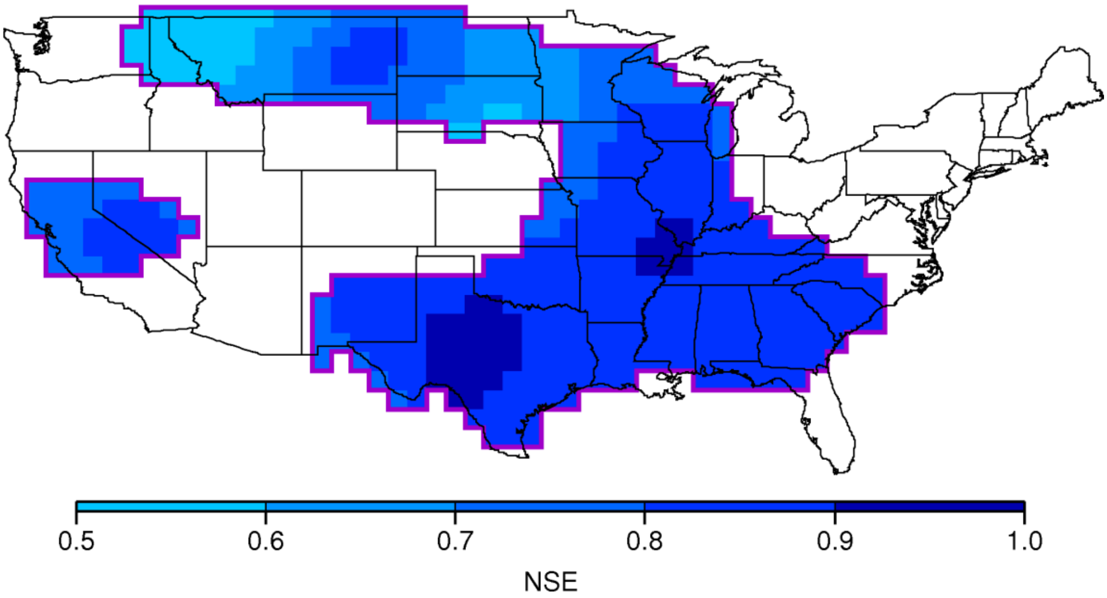

Supplementary Figure S2. Correlation between TWSA and SMS anomaly in the region of model calibration. Correlation coefficients between the time series of GRACE-inferred TWSA and SMS anomaly in the region (purple boxes, also the same light blue region in Fig.3a) where GRACE-inferred TWSA and PHDI values are used to infer the empirical relationship between GHDI and TWSA in Fig. 3b for establishing GHDI. The map was created using the Generic Mapping Tools software package 4.5.12.

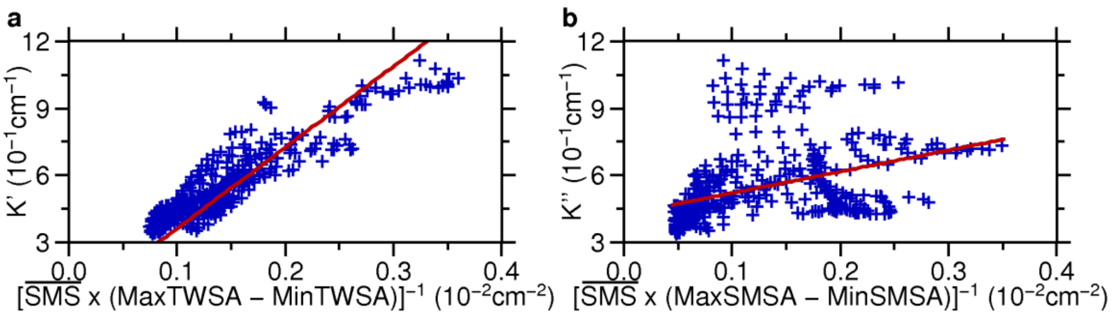

Supplementary Figure S3. Two alternative ways of establishing GHDI. The inferred K' and K'' values vs. values of $\left[\overline{SMS}\times\left(MaxTWSA-MinTWSA\right)\right]^{-1}$ and $\left[\overline{SMS}\times\left(MaxSMSA-MinSMSA\right)\right]^{-1}$ (blue crosses), along with their best fitting linear curves (red lines, Supplementary equation (S13) in (**a**) and Supplementary equation (S15) in (**b**) respectively). Regions of the data calibration is in the light blue area in Fig. 3a.

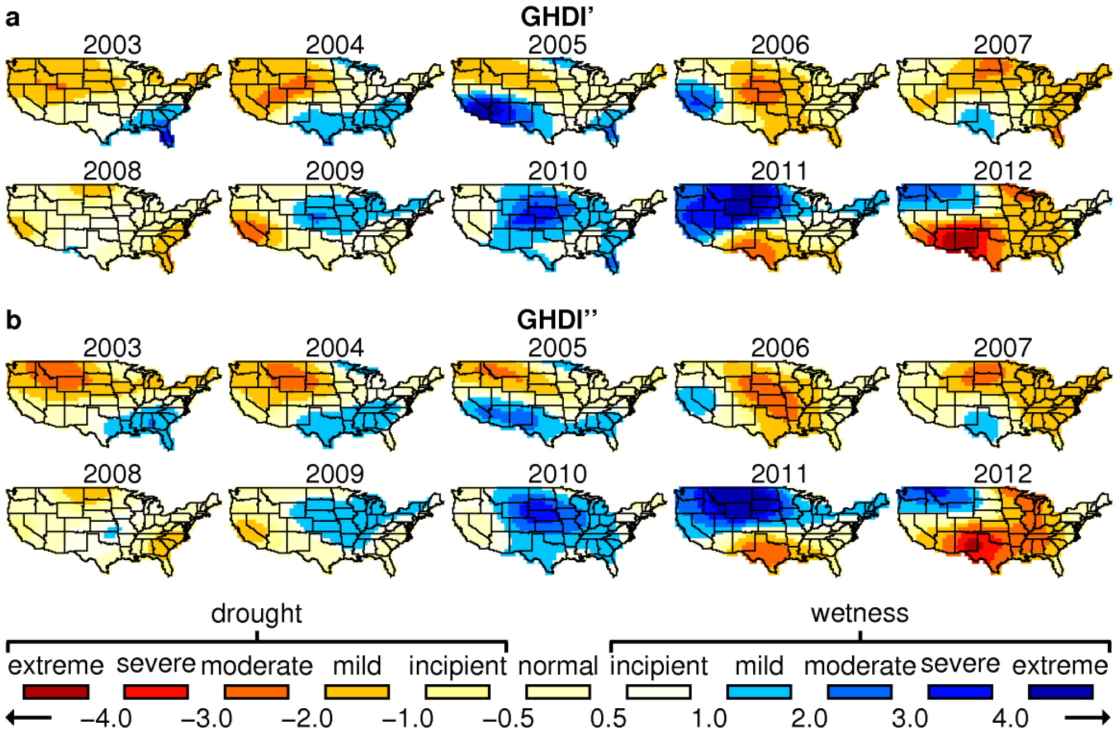

Supplementary Figure S4. Yearly averaged GHDI values established by two alternative ways presented in Supplementary Note 7. Yearly averages of GHDI values in the continental US in the period from 2003 to 2012 with their long-term means removed (the first alternative in (**a**) using Supplementary equation (S13) and the second alternative in (**b**) using Supplementary equation (S15)). The maps were created using the Generic Mapping Tools software package 4.5.12.

Supplementary Table

Supplementary Table S1. Correlation coefficients between relative GRACE-inferred TWS and the inverted ground-based TWS, and energy ratios between soil moisture and groundwater at 12 gray locations labeled in Fig. 2.

Index of selected location	Correlation coefficient	Energy ratio
1	0.71	3.55
2	0.84	0.34
3	0.73	2.96
4	**0.91**	0.94
5	0.71	1.21
6	0.84	0.71
7	0.90	0.06
8	0.84	0.17
9	0.79	1.81
10	0.76	0.98
11	0.61	0.00
12	0.64	0.63

Supplementary Note 1. GRACE data

We use the monthly solutions of the GRACE Level-2 products (Release 05) released at the Center for Space Research, University of Texas at Austin[1,2] (http://isdc.gfz-potsdam.de/index.php), in the period from January 2003 to December 2012 (except for 5 missing months of June 2003, January 2011, June 2011, May 2012 and October 2012) to infer mass changes in the continental US. The Level-2 products provide monthly gravity field estimates in terms of the normalized Stokes coefficients to degree and order 60[1]. These monthly solutions are obtained as gravity change relative to a well-defined reference model. The temporal changes of gravity related to some known geophysical processes have been removed in the Level-2 products from the initial observations, including the solid Earth and ocean tides, selected secular changes, pole-tide effect, atmospheric pressure changes and the ocean response to atmospheric pressure and winds[1-4]. These solutions can thus be attributed to mass changes due to physical processes that were not modeled in the pre-processing of the data.

Uncertainties in GRACE-inferred TWS (error bars in Fig. 2) are estimated based on root-mean-square (RMS) variations over the oceans that have similar latitudes with the continental US (25°N-48°N), but a large distance away from the continent (more than 1,000 km). Considering mass changes in oceans measured by GRACE should be near zero[5] and errors in GRACE data are nearly longitude-independent[6], RMS variations in the remote ocean are used to evaluate the uncertainty in the continental regions with similar latitudes.

Supplementary Note 2. Ground observations of soil moisture and groundwater table and estimate of ground-based TWS

We are able to find 12 locations that have well measurements of soil moisture and 18 groundwater table measurements in close proximity in the period from 2003 to 2012 (Fig. 2).

Daily soil moisture data are retrieved from Soil Climate Analysis Network (SCAN) by the United States Department of Agriculture (USDA) Natural Resources Conservation Service (NRCS) (http://www.wcc.nrcs.usda.gov/scan/). Soil moisture is measured as percentage of soil moisture content at depths of 2, 4, 8, 20 and 40 inches.

Daily groundwater levels are obtained from the United States Geological Survey (USGS) Groundwater Watch (http://groundwaterwatch.usgs.gov/Net/OGWNetworkLTN.asp).

We obtain equivalent water thickness in vadose zone (soil moisture) by summing multiplication products of the measured daily soil moisture with thickness of each soil layer. We then average the results to monthly time series, remove the mean of the monthly time series at each location and obtain monthly soil moisture. For groundwater, we process the data in the same way, i.e., we average groundwater level to monthly time series, remove the mean at each location and obtain monthly groundwater level.

We use two coefficients C_{SM} and C_{GW} to relate the hydrological observations to ground-based TWS at each selected location, specifically,

$$TWS^{g}_{i,j} = C_{SM} \times SM_{i,j} + C_{GW} \times GW_{i,j}, \qquad \text{(S1)}$$

where i is for year, j for month, $TWS^{g}_{i,j}$ monthly ground-based TWS for the j th month of year i, $SM_{i,j}$ monthly soil moisture, $GW_{i,j}$ monthly water level. C_{SM} is introduced to empirically correct potential uncertainties in estimates of soil stratigraphy, regional change of soil deposit and soil moisture in the region, while C_{GW} is equivalently storativity in groundwater well, that is used to convert groundwater level to the associated equivalent water thickness but is lacking in the database. In effect, C_{GW} serves as an average factor for regional change of storativity and groundwater. We obtain C_{SM} and C_{GW} by the least-squares method, fitting TWS^{g} to monthly relative GRACE-inferred TWS. We then calculate the inverted ground-based TWS from Supplementary equation (S1) with the best-fitting coefficients of C_{SM} and C_{GW} and compare it with GRACE-inferred TWS (Supplementary Fig. S1 and Supplementary Table S1, see Supplementary Discussion for details).

Supplementary Note 3. PHDI

PHDI is a hydrological drought index first introduced by Palmer to assess long-term moisture supply in a region (initially, in central Iowa for the period from 1931 to 1957 and in western Kansas for the period from 1887 to 1957)[7]. It is generated monthly and used to indicate the severity of wetness or drought. The index ranges from - 7 to +7, with negative values denoting drought and positive values denoting wetness (Table 1).

Here we briefly introduce its definition. More details can be found in Palmer [7], Alley [8], Karl [9], Wells, et al. [10] and Dai [11].

PHDI is based on the principle of a balance between moisture supply and demand. Man-made changes such as increased irrigation, new reservoirs, and added industrial water use are not included in the computation of this index. PHDI calculations involve four hydrological variables related to soil moisture: evapotranspiration (ET), soil moisture recharge (R), runoff (RO) and moisture loss (L), and their potential values, potential evapotranspiration (PE), potential recharge (PR), potential runoff (PRO) and potential loss (PL). The potential values are weighted by

$$\alpha_j = \overline{ET_j} \times \overline{PE_j}^{-1} \quad \beta_j = \overline{R_j} \times \overline{PR_j}^{-1} \quad \gamma_j = \overline{RO_j} \times \overline{PRO_j}^{-1} \quad \delta_j = \overline{L_j} \times \overline{PL_j}^{-1}, \qquad \text{(S2)}$$

to calculate values of climatically appropriate for existing conditions (CAFEC), where j ranges over the months of a year, $\overline{ET_j}$ is long-term mean of the jth month over the years, and other values have similar meaning.

The CAFEC precipitation is indicated by

$$P_i' = \alpha_j PE_i + \beta_j PR_i + \gamma_j PRO_i + \delta_j PL_i, \qquad \text{(S3)}$$

where i denotes a particular month in a series of months. Then the difference between the actual precipitation and the CAFEC precipitation for each month is

$$d_i = P_i - P_i', \qquad \text{(S4)}$$

which provides soil moisture departure from normal in the study period (in central Iowa for the period from 1931 to 1957 and in western Kansas for the period from 1887 to 1957)[7]. This departure d_i has different meaning in different places and in different time

periods[7]. To make the departure d_i more comparable and meaningful, it is weighted by K_j,

$$K'_j = 1.5\log_{10}\left\{\left[\left(\overline{PE_j}+\overline{R_j}+\overline{RO_j}\right)\times\left(\overline{P_j}+\overline{L_j}\right)^{-1}+2.8\right]\times\overline{D_j}^{-1}\right\}+0.5, \tag{S5}$$

$$K_j = 17.67K'_j \times \left(\sum_{l=1}^{12}\overline{D_l}K'_l\right)^{-1}, \tag{S6}$$

where $\overline{D_l}$ is the mean of absolute values of d in the lth month of a year. The monthly "moisture anomaly index", Z, is defined as

$$Z_i = d_i K_j. \tag{S7}$$

The Z index is considered to express how dry or how wet in a single month without considering historical values. In regard to effect of time and weight by the driest intervals, the drought index is approximated by

$$X_i = \left(0.396i + 2.691\right)^{-1} \times \sum_{t=1}^{i} Z_t, \tag{S8}$$

where t ranges from 1st to ith month in a series of months (there seems a typographical error in equation (20) in page 21 of Palmer [7,8]). To avoid using moisture anomaly of several months ago and considering the drought index X should be invariant for a same drought condition as t increases, Palmer [7] supposed the drought index to be the form of

$$\Delta X_i = X_i - X_{i-1} = 0.333Z_i + cX_{i-1}. \tag{S9}$$

Put values of X_i and Z_i in the driest intervals into Supplementary equation (S9), c is therefore -0.103. The general formula to calculate PHDI is

$$X_i = 0.897X_{i-1} + 0.333Z_i. \tag{S10}$$

We compile PHDI data from NOAA’s National Climatic Data Center (NCDC) in the same study period as GRACE data (ftp://ftp.ncdc.noaa.gov/pub/data/cirs/drd/). NOAA’s NCDC maintains the world's largest climate data archive and provides climatological services and data to every sector of the US economy for users worldwide.

Supplementary Note 4. Soil moisture storage from NLDAS and LSM

We compile soil moisture storage (SMS) data and calculate total soil moisture content from surface to depth of 200 cm (unit: cm)[12], from Phase 2 of North American Land Data Assimilation System (NLDAS-2) simulated from NASA’s Mosaic LSM in the same study period as GRACE data (ftp://hydro1.sci.gsfc.nasa.gov/data/s4pa/NLDAS/). NLDAS-2 is a collaboration project among several groups: NCEP's Environmental Modeling Center (EMC), NASA's Goddard Space Flight Center (GSFC), Princeton University, the NWS Office of Hydrological Development (OHD), the University of Washington, and NCEP's Climate Prediction Center (CPC). NLDAS is a core project with support from NOAA's Climate Prediction Program for the Americas (CPPA). The NASA/GSFC group generated the retrospective Mosaic model simulation. The data have a spatial resolution of 0.125° × 0.125° and a temporal resolution of one month. NLDAS-2 is an offline data assimilation system featuring uncoupled LSMs which are driven by observation-based atmospheric forcing, whose domain covers the continental US, the southern part of Canada, and the northern portion of Mexico (125° to 67° W, 25° to 53° N), and the majority of NLDAS atmospheric forcing data is derived from

the North American Regional Reanalysis (NARR) which features a 32-km spatial resolution and a three-hour temporal resolution[13].

Supplementary Note 5. Nash-Sutcliffe Efficiency

Coefficient of efficiency, defined by Nash and Sutcliffe (Nash-Sutcliffe efficiency, NSE)[14], has been widely used to calibrate and evaluate the performance of hydrological models[15-17]. NSE is defined as follows:

$$NSE = 1 - \frac{\sum_{i=1}^{n}\left(Y_i^{obs} - Y_i^{pre}\right)^2}{\sum_{i=1}^{n}\left(Y_i^{obs} - \overline{Y^{obs}}\right)^2}, \quad \text{(S11)}$$

where Y_i^{obs} is the ith observed value, Y_i^{pre} the ith model predicted value, $\overline{Y^{obs}}$ the mean of observations, and n the number of observations and predicted values. NSE values range from negative infinity to 1 (optimal value). Values between 0 and 1 are considered as acceptable performance for the model, while negative values indicate that the mean of observations is better than model predicted values.

Supplementary Note 6. Support for the assumption of soil moisture contributing most of TWSA in the regions of model calibration

We calibrate GHDI using PHDI values in the regions where PHDI values exhibit good correlation with GRACE-inferred TWSA (light blue regions in Fig. 3a). We choose those regions based on the assumption that soil moisture in the regions contributes most of TWSA and PHDI captures best the terrestrial water storage change there. To present

evidence supporting this assumption, we compare the GRACE-inferred TWSA and SMS anomaly simulated from Mosaic model in the regions of model calibration. The correlation coefficients between the time series of GRACE-inferred TWSA and SMS anomaly are all greater than 0.5 in the regions of model calibration, with 93% of them greater than 0.6 and the highest value reaching 0.92 (Supplementary Fig. S2). These high correlation coefficients support the assumption of soil moisture contributing most of TWSA in the regions of model calibration.

Supplementary Note 7. Two alternative ways of establishing GHDI

We explore two other alternative ways of establishing GHDI and discuss their advantages and disadvantages over our preferred definition in the main text. GHDI is defined as an indicator of the extent of GRACE-inferred TWS anomaly in a region departing from its historical average, i.e., based on $GHDI_{i,j} = K \times TWSA_{i,j}$ (equation (1) in the main text) and $K = a \times \left[\overline{SMS} \times \left(MaxTWSA - MinTWSA \right) \right]^{-1} + b$ (equation (3) in the main text). Here we explore two different ways of defining the scaling parameter *K*.

The first alternative is to define *K* (we name it *K'*) as:

$$K' = a' \times \left[\overline{SMS} \times \left(MaxTWSA - MinTWSA \right) \right]^{-1}, \qquad \text{(S12)}$$

where $\overline{SMS}$ is mean of soil moisture storage (SMS) of the region from 2003 to 2012, *MaxTWSA-MinTWSA* represents the magnitude of historical variation of *TWSA* of the

region in the same time period, and *a'* is proportional constant. The best fitting relationships *K'* are found to be as follows (unit: 10^{-1} cm^{-1}):

$$K' = 36.27 \times \left[\overline{SMS} \times \left(MaxTWSA - MinTWSA \right) \right]^{-1}. \qquad (S13)$$

The difference between this definition and the definition in the main text is that GHDI is directly scaled with TWSA with a constant normalized by $\overline{SMS} \times \left(MaxTWSA - MinTWSA \right)$ in this definition, while GHDI defined in the main text is scaled with TWSA with a constant normalized by $\overline{SMS} \times \left(MaxTWSA - MinTWSA \right)$ and an additional constant (i.e., *b* in equation (2)). The advantage of this method is that magnitude of historical variation of *TWSA* (*MaxTWSA-MinTWSA*) can be separated from definition of *K'*, but the new definition yielded poorer linearity and RMSE in matching the PHDI. The reason is that the linearity constant between PHDI and TWSA can be roughly viewed as consisting of two parts, with one part normalized by mean of *SMS* and historical variation of *TWSA* in a region ($\overline{SMS} \times \left(MaxTWSA - MinTWSA \right)$) and the other part directly to *TWSA* itself. Defining GHDI as $GHDI = K' \times TWSA = a' \times \left[\overline{SMS} \times \left(MaxTWSA - MinTWSA \right) \right]^{-1} \times TWSA$ cannot account for the part that is directly related to *TWSA* itself. Because part of GHDI goal is to resemble PHDI (both for the continuity of PHDI and its extension to other regions), we prefer our original definition to this alterative.

The second alternative is to define *K* (we name it *K''*) as:

$$K'' = a'' \times \left[\overline{SMS} \times \left(MaxSMSA - MinSMSA \right) \right]^{-1} + b'', \qquad (S14)$$

where $\overline{SMS}$ is mean of soil moisture storage (SMS) of the region simulated from Mosaic model from 1979 to 2012 and *MaxSMSA-MinSMSA* the magnitude of historical variation of *SMS* anomaly of the region in the same time period. *a"*, *b"* are proportional constants. The best fitting relationships *K"* are found to be as follows (unit: 10^{-1} cm^{-1}):

$$K" = 9.59 \times \left[\overline{SMS} \times \left(MaxSMSA - MinSMSA \right) \right]^{-1} + 4.25, \tag{S15}$$

The difference between this definition and the definition in the main text is that this definition employs the values of historical variation of *SMS* anomaly in a longer time period from 1979 to 2012 as the normalization factor, while the definition in the main text uses the historical variation of GRACE-inferred *TWSA* from 2003 to 2012. This definition yields poorer linearity and RMSE than the method in the main text, possibly because of errors and lack of accountability of groundwater in Mosaic model. This definition would remove the dependence on GRACE data from *K* and account for the historical variations in a longer time period, but it would require local hydrological history to be known. While these two ways of normalization generate similar GHDIs that both match the observed PHDI, we prefer using GRACE data as normalization and the historical ranges of SMS anomaly in hydrological models as an alternative, as the latter would require detailed and accurate information of the hydrological histories in a region that restricts GHDI to be extended to global scale.

For completeness and comparison, we present yearly averages of GHDI values using the above two alternative definitions in Supplementary Fig. S4a,b respectively.

Supplementary Discussion. Comparison between relative GRACE-inferred TWS and ground observations

We compare the ground measurements to relative GRACE-inferred TWS at 12 different locations (Fig. 2) in the period from 2003 to 2012 (Supplementary Fig. S1). Relative GRACE-inferred TWS exhibits similar trend and annual cycle as the inverted ground-based TWS. Correlation coefficients between two time series are greater than 0.6 at all locations (Supplementary Table S1), with the highest correlation coefficient reaching 0.91 (at location 4 in south region, Fig. 2). These good correlations between the two time series at all 12 locations further support the conclusion that GRACE-inferred TWS represent well the TWS.

The fact that only some appropriate linear combinations of soil moisture and groundwater measurements would fit GRACE-inferred data also lends credibility to estimated relative contributions to TWS change between soil moisture and groundwater. Contributions from soil moisture and groundwater vary from region to region: soil moisture plays the dominant role in TWS change at locations 1 and 3 (contribution from soil moisture greater than 70 percentage, i.e., energy ratio in Supplementary Table S1 greater than 2.33); groundwater greatly contributes to TWS change at locations 2, 7, 8 and 11 (contribution from groundwater greater than 70 percentage, i.e., energy ratio in Supplementary Table S1 smaller than 0.43); and soil moisture and groundwater both

contribute to TWS change at other locations (energy ratio in Supplementary Table S1 between 0.43 and 2.33).

TWS change reflects combined changes of soil moisture and groundwater, and can be estimated based on measurements of soil moisture and groundwater. However, it is in practice challenging to estimate ground-based TWS and compare them to GRACE-inferred TWS, for the following reasons: 1) Most of key background hydrological parameters that are needed to convert soil moisture and groundwater level measurements to equivalent water thickness are not known, including storativity near groundwater well which is needed to convert groundwater table measurement to equivalent water thickness contributed by groundwater, and soil stratigraphy in vadose zone which is needed to convert soil moisture measurement to equivalent water thickness contributed by soil moisture. 2) GRACE observations have a geographical resolution of about 500 km and represent the average effects in a large area, while ground hydrological measurements were made at single locations. 3) Regional changes of hydrological conditions are difficult to assess at various sites.

Although the uncertainties of GRACE-inferred TWS were well established in many studies in the community[5,6,18], comparison between GRACE-inferred and the inverted ground-based TWS may provide another indirect way of further validation. GRACE-inferred TWS exhibits similar trend and annual cycle with the inverted ground-based TWS, at most locations of the continental US and in most time periods. Comparisons of GRACE-inferred TWS and the inverted ground-based observations at

12 locations in the continental US provide further validation of GRACE-inferred TWS, and reveal regional variation of relative contribution of soil moisture and groundwater to TWS change.

Amplitude comparison between GRACE-inferred TWS and inverted ground-based TWS does not have much meaning (although it may provide some insights on the storativity and soil stratigraphy in those locations), as those coefficients are obtained empirically by fitting GRACE-inferred TWS. However, we suggest that the good correlation between (i.e., similar changes within) the time series of these two TWS provides an indirect validation of GRACE-inferred TWS. Through this practice, we can also gain some insight into relative contribution between soil moisture and groundwater to the total TWS change.

Supplementary References

1 Bettadpur, S. Gravity Recovery and Climate Experiment level-2 gravity field product user handbook. Report No. GRACE 327-734, (Center for Space Research, Austin, Texas, 2012).

2 Bettadpur, S. Gravity Recovery and Climate Experiment UTCSR level-2 processing standards document for level-2 product release 0005. Report No. GRACE 327-742, (Center for Space Research, Austin, Texas, 2012).

3 Tapley, B. D., Bettadpur, S., Ries, J. C., Thompson, P. F. & Watkins, M. M. GRACE measurements of mass variability in the Earth system. *Science* **305**, 503-505, doi:10.1126/science.1099192 (2004).

4 Tapley, B. D., Bettadpur, S., Watkins, M. & Reigber, C. The gravity recovery and climate experiment: Mission overview and early results. *Geophys Res Lett* **31**, doi:10.1029/2004GL019920 (2004).

5 Chen, J. L., Wilson, C. R., Tapley, B. D., Yang, Z. L. & Niu, G. Y. 2005 drought event in the Amazon River basin as measured by GRACE and estimated by climate models. *J Geophys Res-Sol Ea* **114**, doi:10.1029/2008JB006056 (2009).

6 Wahr, J., Swenson, S. & Velicogna, I. Accuracy of GRACE mass estimates. *Geophys Res Lett* **33**, doi:10.1029/2005GL025305 (2006).

7 Palmer, W. C. Meteorological drought. Report No. Weather Bureau Research Paper No. 45, (US Department of Commerce, Washington DC, 1965).

8 Alley, W. M. The Palmer Drought Severity Index - limitations and assumptions. *J Clim Appl Meteorol* **23**, 1100-1109 (1984).

9 Karl, T. R. The sensitivity of the Palmer Drought Severity Index and Palmer Z-Index to their calibration coefficients including potential evapotranspiration. *J Clim Appl Meteorol* **25**, 77-86 (1986).

10 Wells, N., Goddard, S. & Hayes, M. J. A self-calibrating Palmer Drought Severity Index. *J Climate* **17**, 2335-2351, doi:10.1175/1520-0442(2004)017<2335:ASPDSI>2.0.CO;2 (2004).

11 Dai, A. Characteristics and trends in various forms of the Palmer Drought Severity Index during 1900-2008. *J Geophys Res-Atmos* **116**, doi:10.1029/2010JD015541 (2011).

12 Mitchell, K. E. *et al.* The multi-institution North American Land Data Assimilation System (NLDAS): Utilizing multiple GCIP products and partners in a continental distributed hydrological modeling system. *J Geophys Res-Atmos* **109**, doi:10.1029/2003JD003823 (2004).

13 Xia, Y. L. *et al.* Continental-scale water and energy flux analysis and validation for the North American Land Data Assimilation System project phase 2 (NLDAS-2): 1. Intercomparison and application of model products. *J Geophys Res-Atmos* **117**, doi:10.1029/2011JD016048 (2012).

14 Nash, J. & Sutcliffe, J. V. River flow forecasting through conceptual models part I—A discussion of principles. *J Hydrol* **10**, 282-290 (1970).

15 Wilcox, B. P., Rawls, W. J., Brakensiek, D. L. & Wight, J. R. Predicting Runoff From Rangeland Catchments - a Comparison Of 2 Models. *Water Resour Res* **26**, 2401-2410 (1990).

16 Legates, D. R. & McCabe, G. J. Evaluating the use of "goodness-of-fit" measures in hydrologic and hydroclimatic model validation. *Water Resour Res* **35**, 233-241 (1999).

17 Gupta, H. V., Kling, H., Yilmaz, K. K. & Martinez, G. F. Decomposition of the mean squared error and NSE performance criteria: Implications for improving hydrological modelling. *J Hydrol* **377**, 80-91, doi:10.1016/j.jhydrol.2009.08.003 (2009).

18 Wahr, J., Swenson, S., Zlotnicki, V. & Velicogna, I. Time-variable gravity from GRACE: First results. *Geophys Res Lett* **31**, doi:10.1029/2004GL019779 (2004).
